# The role of CD38 in HIV infection

**DOI:** 10.1186/s12981-021-00330-6

**Published:** 2021-04-05

**Authors:** Liqi Lu, Jie Wang, Qian Yang, Xiuqiao Xie, Yuanshuai Huang

**Affiliations:** 1 Department of Transfusion, The Affiliated Hospital of Southwest Medical University, 25 Taiping Road, Luzhou, Sichuan 646000 People’s Republic of China; 2 Department of Clinical Laboratory, The First People’s Hospital of Longquanyi District, Chengdu, 201 Third Yihe Group, Longquanyi District, Chengdu, Sichuan 610100 People’s Republic of China

**Keywords:** HIV, CD38 molecule, Abnormal immune activation, ART

## Abstract

The widely-expressed molecule CD38 is a single-stranded type II transmembrane glycoprotein that is mainly involved in regulating the differentiation and activation state of the cell. CD38 has broad and complex functions, including enzymatic activity, intercellular signal transduction, cell activation, cytokine production, receptor function and adhesion activity, and it plays an important role in the physiological and pathological processes of many diseases. Many studies have shown that CD38 is related to the occurrence and development of HIV infection, and CD38 may regulate its progression through different mechanisms. Therefore, investigating the role of CD38 in HIV infection and the potential signaling pathways that are involved may provide a new perspective on potential treatments for HIV infection. In the present review, the current understanding of the roles CD38 plays in HIV infection are summarized. In addition, the specific role of CD38 in the process of HIV infection of human CD4^+^ T lymphocytes is also discussed.

## Introduction

The analysis of CD38 expression on lymphocytes has become an important tool for monitoring patients during HIV infection and has been proposed for use in the follow-up of antiretroviral therapy (ART). CD38 has prognostic utility because it marks the activation of the immune response. CD38 is not only an important prognostic marker but also an active player in HIV infection [1]. There is currently no cure or vaccine for HIV infection, and research on the CD38 molecule will further explain the development and mechanism of HIV infection. This may provide some clues about the clinical treatment of HIV infection. In the present review, the progress of research on the role and mechanisms of CD38 in HIV infection is summarized since an in-depth study of CD38 will provide some ideas about the pathogenesis, disease progression and effective treatment of HIV infection.

## HIV infection

Human acquired immunodeficiency virus (HIV) is a type of RNA virus with two subtypes, HIV-1 and HIV-2, with HIV-1 showing global distribution and HIV-2 being prevalent mainly in West Africa. HIV mainly attacks CD4^+^ T lymphocytes, causing a progressive decrease in the number of CD4^+^ T lymphocytes, resulting in impairment of the body's cellular immune function, and finally to the onset of AIDS. CD4^+^ T lymphocytes [2] and macrophages/monocytes [3] are the primary targets of HIV, but there is some evidence that other members of the hematopoietic system can support HIV infection, dendritic cells [3], natural killer (NK) cells [4] and microglia [5] are included. It was also observed that polymorphonuclear neutrophils depletion [6] and the appearance of a subset of B cells whose function is impaired [7] during the course of HIV-1 infection, but there is still no clear indication that HIV-1 can directly infect polymorphonuclear neutrophils or B cells. But the hematopoietic stem cell is not infectible with HIV, stem cells are highly resistant to HIV infection [8]. HIV infection could lead to various opportunistic infections and tumors, and eventually, it leads to death. AIDS is one of the most serious epidemics ever experienced by humanity, not only because its rapidity and wide spread, but also it is a disease with an extremely high mortality rate.

The characteristic loss of CD4^+^T cells that is thought to play a key role in the development of immunodeficiency. But it is apparently that CD4^+^T cell-directed viral cytopathicity alone cannot explain the course of disease [9]. The cellular immune response to human immunodeficiency virus mediated by T lymphocytes appears to be strong, but does not completely control the infection. Human immunodeficiency virus disrupts this control by infecting key immune cells, thereby weakening the T lymphocyte response. The response of cytotoxic T lymphocytes (CTLs) is important in controlling viral replication [10]. At the beginning of HIV infection, CD8^+^ T cells are stimulated by the virus and activated into cytotoxic T lymphocyte (CTL), which play an important role in the control of HIV through cytotoxic T lymphocyte killing (CTL response). If CD8^+^ T cells do not function effectively, they allow the virus to escape immune control and cause the entire immune system to collapse. Virus-specific CTLs possess a range of antiviral activities, which vary in importance in different infections, including the ability to kill infected cells and to produce cytokines and chemokines [11].

The main immunopathological features of HIV infection include not only reduced numbers and impaired function of CD4^+^ T lymphocytes but also abnormal activation of the immune system. Abnormal activation of the immune system is defined as a state of abnormally elevated activation of the immune system, including lymphocyte subpopulations redistribution, cytokine expression disorders, cell dysfunction and abnormal cell death. Immune activation requires a series of processes such as cell activation, proliferation, apoptosis, and cytokine secretion. A variety of indicators can be used to reflect immune activation levels, such as the expression of activation markers by T cells, B cells, and NK cells, and the levels of chemokines, pre-inflammatory factors, and inflammatory factors in body fluids and lymph nodes [12]. At present, the ratio of CD38 and HLA-DR expression on the surface of T cells has been studied extensively, especially CD38. The prognostic value of T-cell activation increased when immune activation was assessed by co-expression of CD38 with HLA-DR [13] or CD45RO [14], suggesting expression of multiple markers on T cells could be indicative of a hyperactivation status. In the multicenter AIDS cohort study, CD38 expression was a better predictor of HIV disease progression than other markers of immune activation [15].

**Fig. 1:**
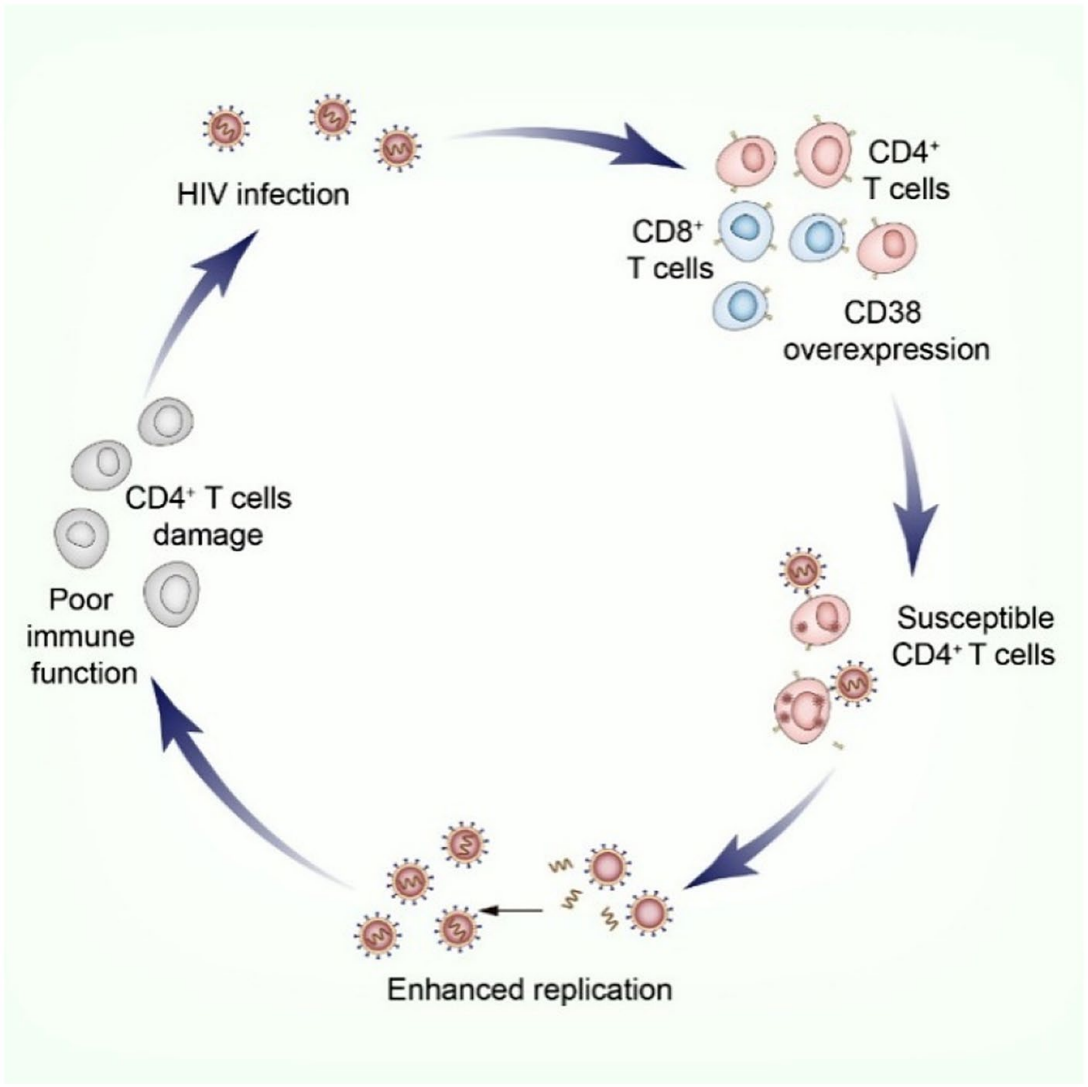
The activation of lymphocytes results in a vicious cycle. Persistent chronic overactivation of CD4^+^ T lymphocytes and CD8^+^ T lymphocytes after HIV infection, with significantly elevated levels of CD38 expression, which may predispose CD4^+^ T cells to HIV infection and further promote HIV replication, and as CD4^+^ T lymphocytes are depleted, the disease continues to progress, forming a cycle

Studies [16] found that CD4^+^ T lymphocytes and CD8^+^ T lymphocytes continue to be chronically overactivated after HIV infection, and CD38 expression levels are significantly increased. The expression of CD38 may predispose CD4^+^ T cells to HIV infection and further enhance HIV replication [17, 18]. As CD4^+^ T lymphocytes are depleted, the disease progresses, creating a cycle that suggests that lymphocyte activation and viral replication are mutually reinforcing (Fig. 1).

## The CD38 molecule

### The distribution and function of the CD38 molecule

Reinherz et al*.* [19] identified the CD38 molecule with a monoclonal antibody in the early 1980s. It was initially considered to be an activation antigen, mainly used for leukocyte classification and phenotypic identification. The gene encoding the human CD38 protein is localized to chromosome 4. The CD38 gene is a single-copy gene that extends over 60 kb, consisting of eight exons and seven introns. As research continued, it was found that the CD38 molecule is a single-chain type II transmembrane glycoprotein comprised of 300 amino acids with a molecular weight of 42–45 kDa, a 21-amino acid cytoplasmic N-terminal domain, a 21-amino acid transmembrane domain, and a 258 amino acid extracellular catalytic and receptor domain [20]. The distribution of CD38 in cells is independent of cell type, but is related to the differentiation and activation state of the cell. The CD38 molecule is widely distributed, and its expression can be detected in peripheral blood T lymphocytes, B lymphocytes, natural killer cells (NK cells), and erythrocytes in healthy humans and in patients with a variety of acute and chronic diseases.

The expression of membrane CD38 can be modulated by certain physiological and pharmacological agents such as cytokines, retinoids, and lectins [21]. In all instances, these agents up-regulate CD38 expression independently of the cell lineage used. In myeloid leukemia cells, CD38 can be induced by retinoids [22, 23]. Cytokines, like IL-15, promotes expansion and survival of CD38^+^/CD8^+^ T cells [24]. This evidence supports that IL-15 may play a role in inducing CD38 expression.

The CD38 molecule has a broad and complex function. For example:

1. Enzyme activity [25]: CD38 was found to share sequence and structural homology with ADPR (ADP-ribosyl) cyclase as early as 1992 [26]. All ten cysteine residues in these two protein molecules are quite conserved, suggesting that they have similar secondary or tertiary structures. Therefore, it is speculated that CD38 should have ADP-ribosyl cyclase activity. It was observed that the CD38 protein molecule also possesses dehydrogenase activity that catalyzes the generation of ADPR from cADPR.
2. Receptor function: CD38 on the surface of the cell membrane has the characteristics of a receptor molecule [27]. Evidence of a receptor function of CD38 should include a well described ligand with a physiological role on defined T cell functions. It has been suggested that the ligand molecule of CD38 is CD31 [28, 29]. CD31 can play a role similar to that of CD38 monoclonal antibody, and its binding to CD38 can cause the release of intracytoplasmic Ca^2+^ and the synthesis and secretion of cytokines.
3. Intercellular and intracellular signal transduction: for example, CD38 is involved in the co-stimulation of T lymphocytes and promotes the growth of B cells in the germinal center to avoid apoptosis [30], it regulates the maturation and differentiation of B cells [31], and it inhibits the growth of normal and leukemia myeloid progenitor cells [32]. Intracellular signal transmission can be accomplished by interacting with other molecular that have transmembrane signaling capabilities. It has been showed that CD38 is associated with TCR, BCR and CD16 of NK cells [33], and the common feature of these molecules is that they have specific signal transduction functions. However, previous experimental results have shown that CD38 can directly transmit signals through its cytoplasmic tail. Because CD38 is spatially related to the SH2 domain of lck through its cytoplasmic tail, and lck is a component of the intracytoplasmic signal chain [34].
4. Cell activation and cytokine production [35]: the combination of CD38 and competitive monoclonal antibodies can transmit activation signals, inducing the activation and proliferation of T cells and NK cells, and it can also mediate the production of IL-1, IL-6, IL-10, IFN-γ, TNF-α, GM-CSF and other cytokines.
5. Adhesion activity [36]: Patterns of cell migration into tissue vary according to the level of expression of CD38, indicating that CD38 is involved in cell-to-cell adhesion. CD4^+^CD45RA^+^ T cells constitutively express low levels of CD38, which mainly migrate from the blood to the lymph nodes, whereas CD4^+^CD45RO^+^ T cells do not express CD38, and preferentially reach lymph nodes via peripheral tissues and the afferent lymph. Activated T cells express high levels of CD38 and tend to migrate to peripheral tissues. And CD38 was found to mediate weak adhesive interactions between leukocytes and human endothelial cells (HEC).

### The role of the CD38 molecule in HIV infection

#### Early findings about the CD38 molecule

The immune system responds rapidly to HIV infection, which results in reduced numbers and the impaired function of CD4^+^ T lymphocytes and abnormal activation of the immune system. Research on abnormal immune activation in HIV-infected patients has been proliferating since the 1990s. Early studies have suggested that the primary hallmark of immune activation in HIV-infected individuals is increased expression of the CD38 molecule on T cells, particularly memory CD8^+^ T cells. CD8^+^ T lymphocytes play an important role in the antiviral response of the body. HIV can induce the formation of specific cytotoxic CD8^+^ T cells after entering the body, which can effectively control virus replication in the early stage of infection [10].

Since CD4^+^ T lymphocytes are the primary receptor cells that are attacked after HIV infection, a decrease in CD4^+^ T cell counts and an increased viral load (number of HIV copies per milliliter of blood) are important signs of disease progression during HIV infection. During early HIV infection, the expression of the CD38 molecule is significantly increased. Using the density of CD38 expression on T cells as a measure of T-cell abnormal activation, lymphocyte activation is one of the possible indirect mechanisms of CD4^+^ T lymphocyte depletion in HIV infection [16, 37, 38].

Currently, systemic chronic immune activation is considered as the driving force of CD4^+^ T-cell depletion and acquired immunodeficiency syndrome (AIDS). However, the causal link between these two phenomena has not been formally established. Binding of HIV to CCR5 or CXCR4 coreceptors on CD4^+^ T cells induces env-mediated signals and activates CD4^+^ T cells by inducing expression of immune markers such as CD25, CD38, CD57, CD69, CD70, and HLA-DR. Activated CD4 cells release soluble factors, including cytokines, which in turn activate CD8 cells [39]. One mechanism by which generalized immune activation may damage the immune system is by providing available targets for HIV replication. Indeed, activation, proliferation and differentiation of naïve and memory CD4^+^ T cells lead to increased CCR5 expression, making these cells more susceptible to infection. The concomitant presence of high levels of CD4^+^ T-cell activation and a virus infecting and killing activated CD4^+^ T cells may help to sustain a vicious cycle, in which infection stimulates activation and activation stimulates infection, which may lead to a severe loss of CD4^+^ T cells [17].

Studies suggested global activation of T cells may explain the more general immunologic dysregulation noted with HIV infection leading to high T-cell turnover, immune senescence, anergy, and finally cell death [12, 40–42]. T-lymphocyte activation is a hallmark of HIV infection that can lead to accelerated T-cell apoptosis [43, 44]. Immune activation contributes more to CD4 decline than the direct effect of HIV [43, 45, 46]. Thus, it is supported the concept that the pathogenic potential of HIV in a given individual is determined both by the level of viral replication and by the ability of a given virus in a given host to cause sustained increases in CD8^+^ T-cell activation [47]. It has been confirmed that viral load is only an indirect contributor to the rate of progression to AIDS, that immune activation predicts changes in CD4^+^ T cells stronger and independent of viral load, and that the effect of anti-retroviral therapy in increasing CD4^+^ T cell counts better correlates with the decrease in immune activation than the suppression of viral load [48–51].

Study [52] have shown that HIV-infected individuals with normal CD4^+^ T cell counts tend to develop to the stage of AIDS more quickly if their CD38 expression levels are higher. In HIV-infected patients with low CD4^+^ T cell levels, their condition can be maintained relatively stable if they have low levels of CD38 expression. This further suggests that the activation of T lymphocytes, on the one hand, contributes to viral multiplication and the expansion of CD4^+^ T cell infection, and, on the other hand, accelerates the destruction of T lymphocyte immune function.

In an earlier period, Liu et al*.* [53] suggested that the level of CD38 expression on the surface of CD8^+^ T cells could predict HIV infection progression and death, but that the level of expression was independent of the HIV viral load in the blood. As research progressed, it was demonstrated that the sustained stimulation of viral antigens after HIV infection in humans puts the immune system in an abnormally high state of activation, and the expression of CD38 on the surface of CD4^+^ T and CD8^+^ T lymphocytes is abnormally elevated and correlates well with plasma viral load [54]. The CD38 molecule can not only reflect the activation status of the lymphocytes, but also reflects the viral load indirectly [13].

#### The advent of the ART era

Since the advent of ART (Antiretroviral Therapy), it has been proven to have positive effects on improving the immune responses, reducing the morbidity and mortality of HIV infected individuals [55], and improving the quality of life of patients [56]. At present, HIV-1 infected patients have a life expectancy that is only marginally shorter than that of HIV-uninfected individuals [57, 58]. ART can suppress HIV replication, reduce the viral load and block viral replication [55, 59], restore CD4^+^ T-cell numbers, reduce microbial translocation, inflammation, and aberrant T-cell activation [60, 61]. The net effect of this is the near restoration of the immune system to pre-infection status and control/prevention of opportunistic infections and other AIDS-associated ailments [62, 63].

It has been suggested that in untreated HIV-infected patients, a significantly elevated expression of CD38 on CD8^+^ T cells is indicative of a poor disease prognosis [64]. And effective antiretroviral therapy is associated with a significant decrease in abnormal immune activation [65, 66]. It has been shown that ART treatment significantly reduces the CD38^+^/CD8^+^ T lymphocytes ratio in HIV-infected patients [67].

A study observed the changes in the expression levels of CD38 on CD4^+^ T and CD8^+^ T cells in the naïve and memory subsets in 25 HIV-infected patients during antiretroviral therapy [68]. The results showed that after 12 months of treatment, the proportion of CD38 expression was reduced in all four cell subsets (CD4^+^ memory cells, CD4^+^ naïve cells, CD8^+^ memory cells and CD8^+^ naïve cells), and the magnitude of CD38 reduction in CD8^+^ T cells was significantly and positively correlated with the increase in CD4^+^ T cells. Another study tracked immune markers and viral load after one year of ART in HIV-infected patients, and the data suggested that an increased frequency of activated CD38^+^/CD8^+^ T cells may contribute to reduced virologic suppression in patients receiving antiretroviral therapy, and that the percentage of CD38^+^/CD8^+^ T cells was negatively, although not significantly, correlated with the CD4:CD8 ratio [69]. It has been suggested that a persistently low CD4:CD8 ratio during long-term effective antiretroviral therapy in HIV-infected patients represents persistent immune dysfunction, which predicts a high risk of non-AIDS morbidity and mortality [70].

Based on various studies in previous years, it has been suggested in recent years that the analysis of circulating CD38^+^ T lymphocyte frequency may become a complementary tool that can be used as a laboratory indicator for monitoring HIV infection trends and treatment response [71–73]. Primary HIV infection (PHI) is the period when the virus begins to replicate rapidly, immediately after an individual is exposed to HIV, and there is a massive increase in the virus in the blood during this period. The viral load is subsequently controlled by the immune system and drops to a modulation point, which is an important indication of the subsequent decline of CD4^+^ T cells and the development of HIV infection [37]. PHI triggers a strong activation of the immune system [74]. A study tested the expression of CD38 on CD4^+^ and CD8^+^ T cells from patients with PHI and found that the proportion of the expression of the CD38 molecule at this time is also an important prognostic parameter for the disease and may be related to disease progression [75]. Appay et al*.* [76] suggested that the timing of initiating antiretroviral therapy during PHI may be critical in reducing the level of immune activation, which has been confirmed in other studies [77]. Early initiation of antiretroviral therapy during the acute infection phase (Fiebig I/II) can reduce activation markers (percentage of mucosal and peripheral CD38^+^/HLA-DR^+^/CD8^+^ T cells) to the levels observed in uninfected individuals. This study demonstrated that initiation of ART in Fiebig I/II completely reversed the initial mucosal and systemic immune activation, whereas patients treated after the acute infection maintained higher mucosal and systemic CD8^+^ T-cell activation after ART initiation. Thus, the timing of initiating treatment during the primary infection is critical for reducing abnormal immune activation levels and may directly affect patient prognosis.

Using the proportion of cells with abnormal immune activation as an indicator of clinical assessment may help in making an early judgment about the HIV-infected patient's response to treatment [78]. The level of CD38 expression affects immune reconstitution after antiretroviral therapy. The magnitude of reduction in the proportion of CD38 expression early in treatment was a predictor of treatment effect. Patients with greater reductions in the proportion of CD38 expression two weeks after the start of treatment took less time to achieve complete viral suppression [79].

Depending on whether they are receiving cART (combined antiretroviral therapy), in HIV-infected patients, CD38^+^ T lymphocytes display different phenotypic and functional responses [80]. Although the CD38 molecule in the peripheral circulation exhibits abnormally elevated levels in T lymphocytes from HIV-infected patients who did or did not receive cART, CD38^+^/CD8^+^ T cells from patients who have already received cART have a higher IL-7 reactivity, which leads to STAT-5 phosphorylation and protection against apoptosis. These changes were not observed in patients who did not receive cART. Such results suggest that cART has a positive effect on the in vivo homeostasis of CD38^+^ T lymphocytes.

#### HICs (HIV controllers) and EUs (HIV-exposed uninfected individuals)

Individuals vary in their susceptibility to HIV infection, and a small number of HIV-infected individuals can spontaneously control the replication of the virus, a group known as “HIV Controllers” (HICs). HICs usually have relatively high CD4^+^ T cell counts and maintain a viral load below the threshold of detection by conventional assays for many years without antiretroviral therapy [81]. The HIC status is related to a variety of factors, including viral defects, cellular factors, and innate immune factors [82], but it is mostly believed that T cells (especially HIV-specific CD8^+^ T cells) play a major role.

As we all know, HICs produce a unique type of CD38^−^/HLA-DR^+^/CD8^+^ T lymphocytes. HICs have been shown to possess HIV-specific CD8^+^ T cells capable of inhibiting in vitro HIV replication [83, 84]. Although HIV-specific CD8^+^ T cells typically express similar levels of two activation markers, CD38 and HLA-DR, and they correlate with viral load in non-HICs [85], they exhibit a unique CD38 low expression, HLA-DR high expression activation phenotype in HICs [83, 86]. The CD38^−^/HLA-DR^+^ phenotype may reflect low levels of activation and thus may contribute to the maintenance of the HIC status [37, 83, 86]. HICs have fewer immune alterations and effector mechanisms (mainly in the peripheral blood) associated with viral control compared to normal progressives. A study exploring the immune properties of gut-associated lymphoid tissue (GALT), the primary target of infection, evaluated the frequency and activation phenotype of T cells in GALT samples from 11 HICs and 15 HIV progressives and showed that T cells in GALT of HICs express HLA-DR but not CD38 [87]. These studies suggest that loss of CD38 expression appears to be associated with better viral control and delayed progression to AIDS.

Another group of individuals who are exposed to HIV but are uninfected are known as HIV-exposed uninfected individuals (EUs) [88], and these individuals have complex antiviral mechanisms in their bodies. These EUs are from different high-risk populations such as sex workers, healthcare workers, hemophiliacs receiving HIV-infected blood, drug users, children of HIV-infected mothers, and individuals in stable relationships with HIV-infected people [88–90]. EUs share the same features as HICs in that both types of T lymphocytes have reduced expression of CD38 [91].

We both observed the HIV-specific CD8^+^ T cells lacking CD38 expression, often along with HLA-DR high expression in HICs and EUs. The low expression of CD38 may reflect the lack of general immune activation, and the expression of HLA-DR may actually characterize T cells with high proliferative potential [83]. This differential expression might be linked to a superior capacity to respond to antigenic stimulation in the absence of nonspecific activation driven by the inflammatory context present in viremic patients, which may associated with better viral control and delayed progression to AIDS.

CD38^−^/HLA-DR^+^/CD8^+^ T cells have been shown to effectively suppress viral replication in autologous CD4^+^ T cells by eliminating HIV-infected cells without the need for exogenous stimulation [83]. This suppressive capacity is not observed in either viremic progressors or in HAART-treated patients and has been associated with an effective loading of perforin and granzyme B in CD8^+^T cells from controllers upon contact with HIV-infected cells [92, 93]. The upregulation of perforin and granzyme B may be related to high levels of expression of the T-box transcription factor T-bet after the in vitro expansion of HIV-specific CD8^+^T cells [94]. The rapid elimination of infected cell targets by ex vivo CD8^+^T cells implies the presence of CTLs with immediate effector functions. Consistent with these results, differentiated effector CD8^+^T cells directed against HIV-1 are found more frequently in HICs compared to HIV-1 progressors [83, 95].

The ability of viral control of CD38^−^/HLA-DR^+^/CD8^+^ T cells is highly correlated with the anti-Gag response, not with Nef- or Env-specific responses [84, 86, 96, 97], which is consistent with earlier studies reporting an association between the anti-Gag CD8^+^T-cell response and viral control [98, 99]. Several studies suggest that Gag-specific CTLs from HICs mediate effective viral control because of their higher functional avidity, that is, the ability to react to lower antigen concentrations and to recognize more epitope variants than those from HIV progressors [100, 101]. A direct contribution to the control of the viral reservoir by the Gag-specific CD8^+^ T-cell response in HICs is suggested by their association with a low HIV reservoir in the central memory CD4^+^ T cells of HICs carrying HLA-B57/27 alleles [102].

It has been demonstrated that CD38^−^/HLA-DR^+^ cells are characterized by low expression, high viability, proliferative capacity and high cytotoxicity in vitro compared to CD38^+^/HLA-DR^+^ cells, and that CD38^−^/HLA-DR^+^ cells are preferentially generated at low viral antigen concentrations [103]. Induction of this protective CD8^+^ subtype may be critical for HIV vaccine development. A separate study has found that CD38 is highly expressed in central memory CD4^+^ T cells in HIV-infected patients receiving ART for more than 5 years [104], which we know may play an important role in the long-term presence of HIV in the body. The findings of this study suggest that memory CD38^+^ /CD4^+^ T cells perpetuate the virus in HIV-infected patients receiving long-term ART, which may provide a potential target for addressing the persistence of HIV in humans.

#### Infected children and pregnant women

In a series of previous studies, it was not difficult to conclude that increased levels of CD38^+^/CD8^+^ T cells in adults are a strong indicator of disease progression after HIV infection. However, some studies have found conflicting data in children with HIV infection. Freguja and colleagues [105] reported a study that evaluated the relationship between viral load and immune activation in HIV-1-infected children. The study showed a strong relationship between viral load and immune activation, suggesting that viral load induces a rise in immune activation. Romeiro et al*.* [106] found that a higher percentage of activated CD8^+^ T cells was present in all HIV-infected children regardless of disease progression, and that CD8^+^ T cell activation was not associated with the viral load or the percentage of CD4^+^ T cells. Therefore, it was concluded that the expression of CD38 in CD8^+^ lymphocytes from HIV vertically infected children was not associated with disease progression, and these immunological parameters could not be applied to the prognostic assessment. The view of this study is contrary to that of studies in adult HIV-infected individuals, and it is speculated that it may be related to the immature immune system in children, for reasons that will require more direct studies to show. It has been reported that CD38 is expressed early in hematopoietic cells, with downregulation during the cell maturation process and re-expression upon cell activation. This cyclic nature of CD38 expression during lymphopoietic ontogeny may explain the expression of CD38 in HIV-infected children [106].

And in children accepted ART, immune reconstitution is different from that in adults. It involves mainly naïve cells, due probably to more efficient thymopoiesis, and some children have a gain in CD4 cells despite persistent detectable viraemia [107–109]. Persistence of chronic immune activation has been described in children with such a discordant response to ART. In children with this discordant response to therapy, there is a persistence or even an increase in immune activation [110].

The previous study shown that the total CD8^+^ lymphocytes may not be altered by pregnancy, but activated CD8^+^ T lymphocyte counts in HIV-positive pregnant women (CD38^+^/CD8^+^ T and HLA-DR^+^/CD8^+^ T lymphocytes) were found to be lower than in HIV-positive women who were not pregnant, and the counts were also reduced in the second and third trimesters [111]. Based on this study, it can be inferred that pregnancy has a significant inhibitory effect on CD8^+^ T lymphocytes immune activation during HIV infection.

Another study shown that pregnancy contributes to the activation of peripheral CD8^+^ T cells and increase in pro-inflammatory cytokines [112]. Immune activation has been documented in both pregnant and non-pregnant HIV-infected women [113–116]. In a 2017 paper in Nature Reviews Immunology, Mor et al*.* [117] described the occurrence of three normal successive immunological stages during gestation. They further proposed in this same paper that inflammation induced by HIV infection could disrupt this ordered sequence and increase the risk of fetal damage and premature birth. Studies have shown that the heightened immune activation observed in pregnant HIV infected women resulted from the combined effects of both pregnancy and HIV-mediated inflammation, which in turn promoted higher HIV viral loads [113, 116].

#### AIDS complications and co-infections

HIV attacks the immune system throughout the body and can cause numerous complications in various parts of the body. For example, HIV can cross the blood–brain barrier and may lead to HIV-associated neurocognitive disorders (HAND) [118]. In some patients with HIV-associated neurocognitive deficits (HAND), astrocyte surface levels of CD38 are significantly elevated [119], presumably regulated through the MAPK signaling pathway and the transcription factor NF-κB. Using HIV-RNA (VL) 20 copies/ml as a cut-off value to distinguish viremia from non-viremia, a study found that CD38 expression of CD4^+^ T lymphocytes or CD8^+^ T lymphocytes in both peripheral blood and cerebrospinal fluid was different between the two groups. The T cell activation/apoptosis markers in the cerebrospinal fluid were negatively correlated with the central nervous system permeability score (CPE), suggesting its importance for restoring immune function therapy [120]. These findings suggest that CD38 is a potential marker to distinguish between two patient subgroups, with and without cerebrospinal fluid viremia.

The finding of an elevated percentage of circulating CD38^+^ T cells should warrant clinicians to consider the possibility of the transmission of HIV infection to the nervous system in order to intervene earlier to prevent progression. In addition, in patients with oral lesions due to HIV infection [121], median CD38 levels on the surface of T lymphocytes in peripheral blood were found to be significantly higher in patients with oral mucosal lesions than in patients without oral lesions, and median CD38 levels were also significantly higher in patients with gingivitis and periodontitis, suggesting that elevated CD38 expression levels in peripheral blood in HIV-positive patients are associated with oral lesions.

Significantly elevated expression of CD38 was observed in both CD4^+^ and CD8^+^ T cells in HIV/HCV co-infected patients compared to mono-infected patients and healthy controls. There was a direct correlation between the expression of CD38 in CD4^+^ T cells and CD8^+^ T cells, suggesting a synergistic increase in the level of immune activation in both T cell subsets. Despite effective mediation of HIV suppression by ART, HIV/HCV co-infected patients still have high expression levels of CD38. When HCV replication was suppressed by IFN-α and ribavirin treatment, CD38 expression was decreased, suggesting that immune activation is also associated with a high HCV viral load in co-infected patients [122]. However, in another study [123], it was found that in HIV/HCV co-infected patients, HIV plays a key role in determining host immune activation, whereas the role of HCV needs further investigation.

### Expression of the CD38 molecule in other diseases

In addition to co-infection, similar results have been reported in other types of viral infections, such as HBV infection [124]. It was found that CD4^+^ T cell counts and CD8^+^ T cell counts in the peripheral blood were decreased and that the proportion of CD38^+^/CD8^+^ T cells was increased in patients with chronic hepatitis B, which was significantly higher than in HBV carriers and healthy controls. In patients with hepatitis B treated with and responding effectively to adefovir, the proportion of CD38^+^/CD8^+^ T cells decreased significantly, accompanied by a decrease in viral load. CD38^+^/CD8^+^ T cell counts fluctuated more in patients who failed to respond to treatment. This study demonstrates that abnormally activated CD8^+^ T cells in chronic HBV infection can be partially reversed by antiretroviral therapy. Measuring the expression level of CD38 and other molecules on PBMC in HBV-infected patients helps to objectively reflect the state of cellular immune function in these patients, and at the same time, has a certain reference value for the patient's disease regression and the judgment of the treatment effect.

CD38 also plays an auxiliary role in the assessment of malignant tumor disease and prognosis. It has been shown that the expression of CD38 in epithelial ovarian cancer (EOC) is higher than in normal tissues, and high CD38 levels are associated with prolonged disease-free survival and a significant increase in overall patient survival [125]. CD38 contributes to the regulation of antitumor immunity and can be used as a prognostic biomarker and potential immunotherapeutic target, which suggests a potential application of CD38 as a tumor marker.

Several hematologic malignancies also express varying levels of CD38 [126–128], such as multiple myeloma (MM), chronic lymphocytic leukemia (CLL), non-Hodgkin's lymphoma (NHL), acute lymphoblastic leukemia (ALL), and acute myeloid leukemia (AML). In hairy cell leukemia, elevated levels of CD38 expression are a marker of a poor prognosis [129], and CLL patients with high CD38 expression also have more aggressive clinical behaviors and a less optimistic prognosis [130]. A study [131] analyzed the CD38 expression levels of acute T-lymphocytic leukemia in children and showed that 97.9% of T-ALL patients were positive for CD38, and T-ALL patients had significantly higher CD38 expression than B-ALL and AML patients. The high CD38 expression group had a worse early treatment response than the low CD38 expression group. Other studies have suggested that CD38 is only minimally expressed on normal lymphocytes or bone marrow cells, but is highly expressed on the surface of myeloma cells, so the CD38 molecule may be a new target for MM therapy [132, 133].

The CD38 molecule has also been associated with the development of several autoimmune diseases. The reason for this may be that it catalyzes the metabolism of two calcium messengers, cyclic adenosine diphosphate ribose (cADP) and nicotinic acid adenine dinucleotide phosphate (NAADP), which are involved in intracellular calcium signaling and the modulation of immune cell function [134]. For example, CD38 is highly expressed in the blood, CD3^+^ and CD56^+^ subpopulations of patients with rheumatoid arthritis, and the percentage of CD38^+^ cells is significantly correlated with RF levels [135]. Therefore, some researchers have suggested that CD38 may be a potential target for RA disease blockade [136].

## Future perspectives

As the research proceeds further, increasing numbers of researchers are suggesting that abnormal immune activation may be an important factor in mediating immunodeficiency and disease progression, independent of HIV replication. Since CD38 expression is intrinsically correlated with CD4^+^ T cell counts, but independent of whether CD38^+^ lymphocytes are in a circulating state, it may be important to suggest that CD38 expression is independent of the cell cycle and thus may itself be involved in pathogenic mechanisms [137]. Whether markers of immune cell activation, particularly the CD38 molecule [38], reflect or participate in other processes leading to HIV infection progression, remains to be elucidated. Even though CD38 expression is highly correlated with T cell activation, its function in this process is still not fully understood.

The expression of CD38 on T cells strongly predicts the risk of HIV infection progression, but it is not known whether CD38 is a marker or receptor for immune dysfunction. The CD38 molecule has strong immunomodulatory functions [138]. Soluble CD38-regulated dendritic cells produce IL-10 and TGF-β, thereby decreasing the cellular immune activity of CD4^+^ lymphocytes [139]. Based on these findings about the CD38 molecule, most investigators believe that sustained immune activation of CD8^+^ T cells may be a marker of progression of HIV infection [111]. Many investigators have suggested that CD38 on CD8^+^ T cells alone predicts HIV infection progression independent of viral load and CD4^+^ T cell count, but others have suggested that the marker itself is not sufficiently sensitive or specific [140].

However, in previous studies, we learned that CD38 is enzymatically active and it is a well-known exonuclease that catalyzes the conversion of nicotinamide-adenine dinucleotide (NAD) to ADPR, cADPR, and NAADP. These "second messengers" can regulate T cell function. Several studies [141–144] concluded that the relationship between CD38 and the development of HIV infection might be attributable to the enzyme activity of CD38.

Juan et al*.* [145] also suggested that the possible mechanism of CD4^+^ T cell depletion was due to the enzymatic activity of CD38. These researchers found that increased CD38 catalytic activity may reduce cytoplasmic NAD in CD4^+^ T cells, leading to a chronic Warburg effect (i.e., aerobic glycolysis). This metabolic state was discovered by Warburg in malignant tumor cells in 1956, and it is essentially a marked enhancement of glycolytic pathways under aerobic conditions, as evidenced by increased glucose uptake and increased lactate production [146], which would reduce mitochondrial function.

**Fig. 2:**
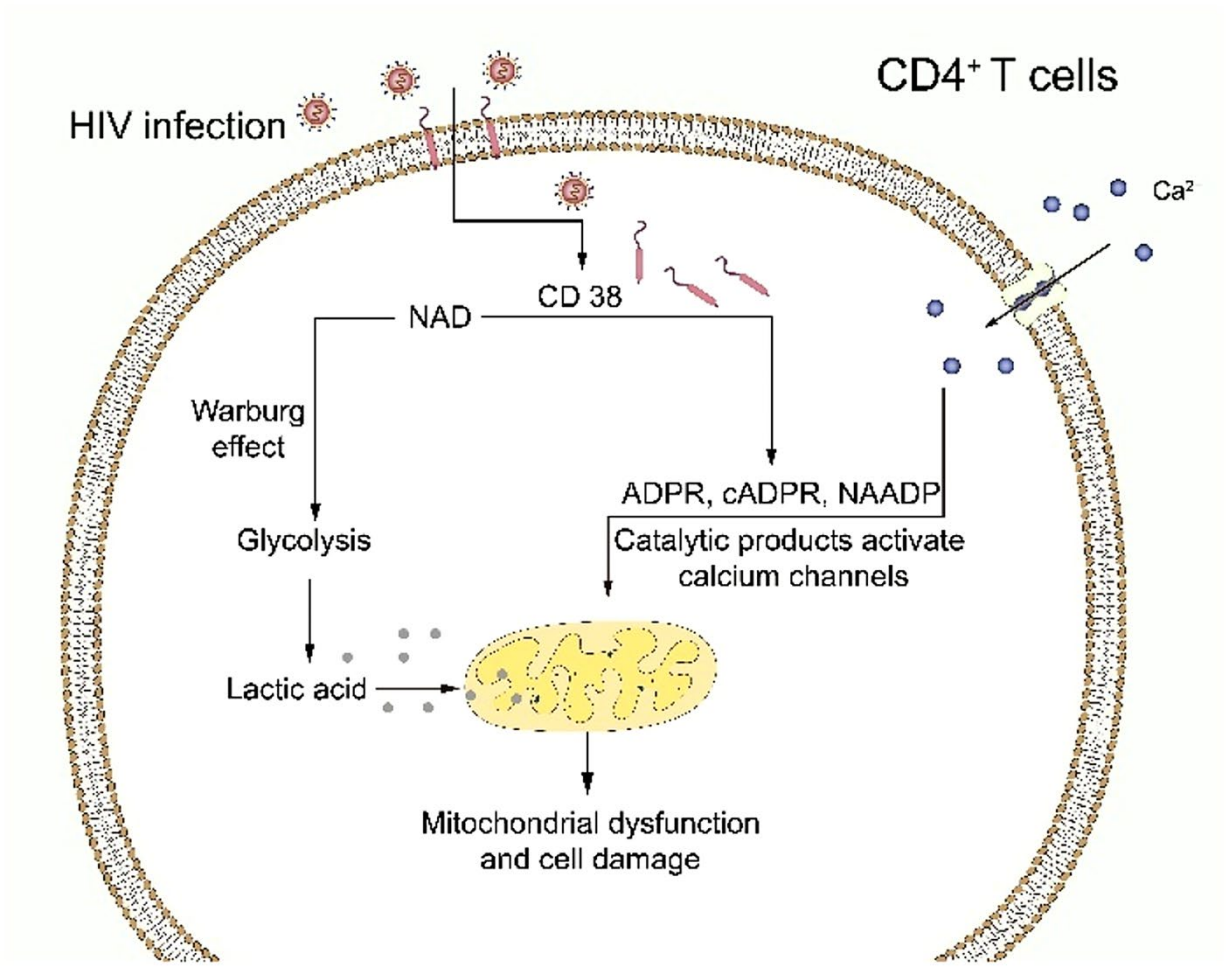
Enzymatic activity of CD38 molecules leads to internal alterations in CD4^+^ T cells. CD38 catalyzes the conversion of NAD to ADPR, cADPR, and NAADP, which reduces cytoplasmic NAD in CD4^+^ T cells, leading to a chronic Warburg effect that decreases mitochondrial function. The catalytic products of CD38 can activate calcium channels and increase cytoplasmic Ca^2+^ concentrations, further reducing mitochondrial integrity. Depletion of cytoplasmic NAD promotes the Warburg effect and continues to affect mitochondrial function and integrity. Elevated cytoplasmic Ca^2+^ concentrations further increase mitochondrial stress, which ultimately leads to decreased CD4^+^ T cell survival

At the same time, ADPR and cADPR, as catalytic products of CD38, can activate calcium channels and increase cytoplasmic Ca^2+^ concentrations, further reducing mitochondrial integrity and thereby reducing CD4^+^ T cell viability and regenerative capacity. Chronic increased activity of CD38 in the memory CD4^+^ T cell environment may increase NAD conversion, leading to cytoplasmic NAD depletion, which in turn can promote the Warburg effect and affect mitochondrial function and integrity. In addition, elevated concentrations of cytoplasmic Ca^2+^ would further increase mitochondrial stress, ultimately leading to decreased survival of CD4^+^ T cells (Fig. 2).

If the above hypothesized pathogenic mechanism which kills CD4^+^ T cells independently of viral infection can be proven experimentally, it will reveal new therapeutic targets that may be used as a strategy for antiretroviral therapy in the future.

In fact, abnormal immune activation has emerged as a potential therapeutic target. Researchers have attempted to reduce activation levels by applying immunosuppressive agents or modulators in order to reduce immune damage [147]. However, since most clinical trials aimed at reducing aberrant immune activation only included a limited number of patients or showed effects on immune activation biomarkers only, these studies have not been able to assess any impact of suppressing or reducing immune activation levels on clinical outcomes.

AIDS caused by HIV infection is a disease of the immune system. The CD38 molecule acts as a transmembrane glycoprotein on the cell surface and it is closely related to the development of several diseases. CD38 is barely expressed on the surface of lymphocytes in HIV-negative adults. CD38 has been widely recognized as a potential prognostic marker for HIV-positive individuals prior to antiretroviral therapy (ART) and its expression level predicts a downward trend in viral load after initiation of therapy. The detection of CD38 provides a reliable real-time complementary test for long-term viral load monitoring in HIV-infected patients [148]. However, the mechanism of the CD38 molecule in HIV infection has been poorly reported and needs to be further investigated in view of the broad and complex role of this cell surface molecule.

## Conclusion

The expression of the CD38 molecule is widely distributed, and its presence in cells is related to their differentiation and activation state. The expression of CD38 can be detected in T-lymphocytes, B-lymphocytes, natural killer cells (NK cells) and erythrocytes in the peripheral blood of healthy humans or patients with various acute/chronic diseases [21]. The functions of the CD38 molecule are extensive and complex, including enzyme activity, intercellular signal transduction, cell activation, cytokine production, receptor function and adhesion activity [149]. After HIV infection, T lymphocytes continue to be chronically over-activated, and the expression level of CD38 is significantly increased. CD38 not only reflects the activation status of lymphocytes but also indirectly reflects the viral load. ART significantly reduces the proportion of CD38-positive lymphocytes in HIV-infected patients, and its count and percentage reflect the viral load in the body, which is an indicator of the antiviral effect of the treatment [150]. The results of this review suggest that CD38 is not only an indicator of elevated post-infection, but also an active player in HIV-1 infection. To sum up, expression of CD38 may predispose cells to HIV infection and further enhance HIV replication. With the depletion of CD4^+^ T lymphocytes, the disease continues to progress, creating a vicious cycle. However, the knowledge about CD38 in HIV infection is currently inconclusive as to whether it is a player independent of viral replication that mediates immunodeficiency and disease progression or is a by-product of T cell activation.

Various studies have proven the necessity and feasibility of using the CD38 molecule as a research object in HIV infection research. It is believed that with in-depth research using the CD38 molecule as the target, we can further understand the series of changes in the body after HIV infection, or further reveal the pathogenesis of HIV infection, and contribute to the development of a vaccine or find new treatment methods.

## Data Availability

Not applicable.

## Abbreviations

ALL: Acute lymphoblastic leukemia
AML: Acute myeloid leukemia
ADPR: Adenosine diphosphate ribose
AIDS: Acquired immune deficiency syndrome
ART: Antiretroviral treatment
BCR: B cell receptor
cADPR: Cyclic adenosine diphosphate ribose
cART: Combined antiretroviral therapy
CD: Cluster of differentiation
CPE: Central nervous system permeability score
CLL: Chronic lymphocytic leukemia
cADP: Cyclic adenosine diphosphate ribose
CTL: Cytotoxic T lymphocyte
EOC: Epithelial ovarian cancer
EUs: HIV-exposed uninfected individuals
GM-CSF: Granulocyte macrophage-colony stimulating factor
GALT: Gut-associated lymphoid tissue
HBV: Hepatitis B virus
HCV: Hepatitis C virus
HEC: Human endothelial cells
HICs: HIV controllers
HIV: Human immunodeficiency virus
HAND: HIV-associated neurocognitive disorders
HLA: Human leukocyte antigen
IFN-γ: Interferon-gamma
IL: Interleukin
MM: Multiple myeloma
NK: Natural killer cells
NAD: Nicotinamide-adenine dinucleotide
NAADP: Nicotinic acid adenine dinucleotide phosphate
NHL: Non-Hodgkin's lymphoma
PHI: Primary HIV infection
RF: Rheumatoid factor
TCR: T cell receptor
TGF-β: Transforming growth factor-beta
TNF-α: Tumor necrosis factor-alpha
